# Lab on a Chip for the Colorimetric Determination of Nitrite in Processed Meat Products in the Jordanian Market

**DOI:** 10.3390/mi10010036

**Published:** 2019-01-07

**Authors:** Mohammad F. Khanfar, Nour J. Abu Eisheh, Loiy Al-Ghussain, Ala’aldeen T. Al-Halhouli

**Affiliations:** 1 Department of Pharmaceutical and Chemical Engineering, School of Applied Medical Sciences, German Jordanian University, Amman 11180, Jordan; Mohammad.Khanfar@gju.edu.jo (M.F.K.); N.AbuEisheh@gju.edu.jo (N.J.A.E.); 2 NanoLab, Mechatronice Engineering Department, School of Applied Technical Sciences, German Jordanian University, Amman 11180, Jordan; loui.essam@hotmail.com

**Keywords:** Lab on a Chip, nitrite, griess reaction, processed meat, microfluidic

## Abstract

Nitrite and Nitrate have been used extensively as additives in various meat products to enhance flavor, color, and to preserve the meat from the bacterial growth. High concentrations of nitrite can threat human health since several studies in the literature claim that nitrite is associated with cancer incidences, leukemia, and brain tumors. Therefore, it is vital to measure the nitrite concentrations in processed meat products. In this study, an in-lab miniaturized photometric detection system is fabricated to inspect the nitrite concentration in processed meat products in Jordan. The analytical performance of nitrite detection is evaluated based on three key statistical parameters; linearity, limit of detection, and limit of quantitation. Respectively, for the fabricated system, the three values are found to be equal to 0.995, 1.24 × 10^−2^ ppm, and 4.12 × 10^−2^ ppm. Adherence to Beer’s law is found over the investigated range from 2.63 ppm to 96.0 ppm. The developed system is utilized for photometric detection of nitrite in processed meat products available in the Jordanian market like pastrami, salami, and corned beef. In all of the analyzed samples, the nitrite content is found to be lower than 150 ppm, which represents the maximum allowable nitrite limit.

## 1. Introduction

Nitrite anion is a triatomic ion with two oxygen atoms and one nitrogen and an overall charge of −1. With that structure, the nitrogen atom has an oxidation state of 3+ which is not the most stable oxidation state for nitrogen, therefore, nitrite does not exist naturally except in reductive environments that facilitate the reduction of nitrate (where nitrogen has the most stable 5+ oxidation state). As a result, nitrite naturally exists in relatively very low concentrations in nature. Therefore, nitrites are usually synthesized chemically rather than being extracted naturally [1].

Nitrite has been used for decades as a food additive where it is added as sodium (coded E-250) or potassium salts (coded E-249). Sodium nitrite is one of the most important additives used in the meat industry. Nitrite serves many purposes as an additive where it is a bacteriostatic and sporostatic. As a bacteriostatic, it prevents the growth of bacteria specifically Clostridium botulinum which causes botulism while as a sporostatic, it inhibits the growth of spores. Without this control of microorganisms, the shelf life of the meat decreases due to deterioration of the product and loss of quality, which has negative economic implications. The minimum amount of added nitrite needed to secure a safe shelf life of cured meat is 25 ppm. However, higher concentrations that vary between 25 ppm and 125 ppm are employed since the saltiness of cured meat depends on taste and acceptance of the consumers [2,3,4]. The microorganisms may also be responsible for human illness after consumption of the spoiled meat. Moreover, nitrite is used in meat is to enhance its aesthetics or attractiveness. To do so, nitrite is converted to nitric oxide which associates with the myoglobin in the meat. The myoglobin accounts for the natural red pigment in uncured meat. The resulting nitric oxide myoglobin gives a deep red color, which turns to pink when heated in the smoking process of meat [5,6,7,8].

Furthermore, nitrites also impede the rancidity of meat products, which may cause off-flavors and off odors. Lastly, the nitrite additives also have antioxidant characteristics [5,6,7,8]. Despite these numerous advantages, high concentrations of nitrites have adverse health effects making them closely monitored and regulated. According to the Directive No. 95/2/EC of the European Parliament and of the Council of February 20, 1995 on food additives other than colors and sweeteners, both sodium nitrite and potassium nitrite are allowed with maximum concentrations of 150 mg/kg (150 ppm) in meat products [9]. In high concentrations, nitrite reacts with ferrous ion Fe^2+^ in hemoglobin and oxidizes it to form methemoglobin with ferric ion Fe^3+^. Methemoglobin does not transport oxygen as well as hemoglobin and would cause further health complications such as blue baby syndrome, fast heart rate, and breath shortness [10]. Moreover, several studies in the literature claim that there is a connection between nitrite and cancer and tumors in adults and children.

The detection of nitrite in samples of different origins such as natural waters, processed meats, and biological samples like urine and blood plasma has been carried out by different research groups [11,12,13,14,15,16,17]. The ion could be detected by the three key instrumental methods, namely ion chromatography, electrochemistry (both potentiometry and voltammetry), and spectrometry. Ion chromatography can be used to detect the nitrite anion by suppressed conductivity detection or by UV absorption. Ion chromatography is often used to detect nitrite in meat samples as it takes a short retention time, is highly sensitive, and requires small sample volumes [11,12].

At the same time, the potentiometric determination of nitrite ion requires the utilization of the NO_2_^−^ ion selective electrode, which has an internal standard nitrite solution with constant composition confined by porous membrane. Once the nitrite ion is inserted in the test solution, potential difference across the membrane is established where it is proportional to the concentration of the nitrite in the test solution. For the voltammetric quantitation of nitrite, a potential is applied on a sensing electrode where the potential value is carefully set to the voltages at which nitrite electrolysis takes place, so nitrite could be reduced to ammonia or oxidized to nitrate. In both cases, the current produced from the electrolysis process is correlated to nitrite concentration [18,19,20,21].

**Figure 1:**
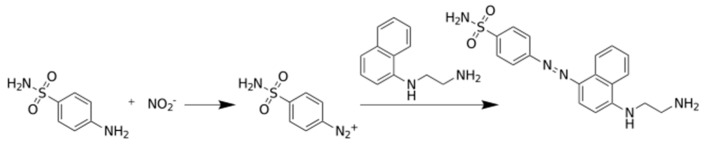
The Griess reaction for the photometric determination of nitrite ion.

Photometric determination of nitrite is the standard method recommended for nitrite detection, it is based on Griess reaction [22,23,24]. According to the reaction, nitrite enters a series of reactions as a limiting reactant, and these transformations end with colored compound where the absorption of the nitrite depends on the initial nitrite concentration. The chemical transformation is shown in Figure 1.

The detection of nitrite by miniaturized systems has been performed by different research groups across the world [14,16,17,25,26]. Key examples on the utilized systems are listed in Table 1. Miniaturization of detection systems has attracted a lot of attention recently since it is accompanied by portability, running cost efficiency, simplicity, and convenience in addition to the minimization of chemicals and reagents consumption, since the desired analyses can be performed completely and successfully with submillimeter amounts of fluids [13,27,28,29,30,31,32,33,34]. The use of Lab on Chip (LOC) in the miniaturized detecting systems has been investigated extensively by researchers. In this study, the nitrite detection is performed using LOC technology that can ensure the miniaturization of the chemical procedures required for the nitrite detection. This study was performed with three main objectives in mind; fabrication of microfluidic system presented by the lab on a chip model, utilization of the fabricated model for nitrite detection, and finally validation of the employed analytical model by the key statistical parameters such as linearity, limit of detection, limit of quantitation and recovery. Statistical validation of the employed system is based on the method external standard method known also as the calibration curve, as will be shown in the next sections.

## 2. Experimental Methodology

### 2.1. Chemicals Preparations

The detection of nitrite process requires several chemicals which are shown in Table 2.

High Performance Liquid Chromatography (HPLC) grade water from UltraMax 372 Yonglin Water Purification System, Anyang, South Korea is used for the preparation of the tested solutions. Phosphate buffer solution with pH of 2.0 is used in this study as the working solution, which is prepared by mixing phosphoric acid and dihydrogen sodium phosphate in appropriate amounts where 0.1 M NaOH aqueous solution is used to adjust the pH of the working solution. Buffer solutions of Benzensulfanylamide (S.A.) and N-1-naphthylethylenediamin dihydrochloride (NEDA) with concentration of 1.0 mM is mixed with potassium nitrite which is prepared with a concentration of 0.5 mM in order to get the required solution with the desired pink color. Moreover, several samples of the standard solutions with different concentrations are prepared by serial dilution where the fabricated detection system is used to measure the absorbance of the solutions.

### 2.2. Microfluidic Platform Design and Fabrication

**Figure 2:**
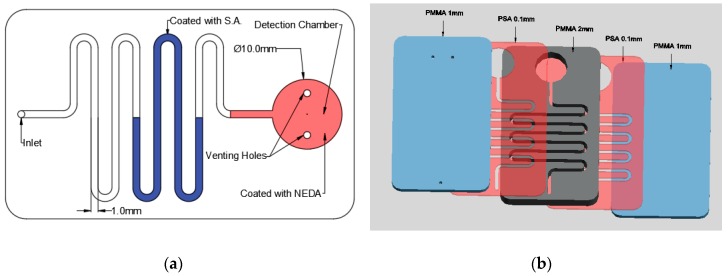
Nitrite detection microfluidic chip: (**a**) chip design and (**b**) the layers of the microfluidic chip.

As aforementioned, the nitrite detection is done using LOC technology; therefore, it is important to obtain the suitable microfluidic chip. In this study, a simple microfluidic chip is designed and fabricated in the laboratory. Figure 2 shows the design of the fabricated microfluidic chip. The microfluidic chip consists of a detection chamber with a diameter of 10 mm with two venting holes to prevent the formation of positive pressure inside it which will block the movement of the fluid. Moreover, the detection chamber is connected to a channel with 2 mm depth and 1mm width where it has an inlet hole at the end with 1mm diameter for fluid injection as shown in Figure 2a.

The microfluidic chip is fabricated using polymethyl methacrylate (PMMA) plastic from Moden Glas, Bangkok, Thailand and transparent pressure-sensitive adhesive (PSA) with 100 µm thickness from FLEXcon (Spencer, MA, USA). As shown in Figure 2b, the chip consists of two 1 mm PMMA layers, one 2 mm layer and two PSA layers. The channels and detection chamber are fabricated on the 2 mm PMMA layer as well as the two PSA layers. On the other hand, the bottom 1 mm PMMA has no holes or features where it is used as a cover while the top 1 mm layer has one inlet hole and two venting holes. Bodor CO_2_ laser cutter (Bodor, Jinan, China) with 90 W maximum power is used for the fabrication of the microfluidic channels and chamber. The PMMA layers are then cleaned and washed to get rid of the suspended dust and particles that could affect the bonding effectiveness. After the cleaning process the PMMA layers are aligned and then bonded together using the PSA layers. After placing the PSA layers between the PMMA layers, it is placed in a manual press machine for one day to ensure the maximum adhesion between the layers.

The detection of nitrite requires the suspension of S.A. and NEDA; therefore, the channel in the microfluidic chip is coated with S.A while the detection chamber is coated with NEDA using a micropipette. S.A. and NEDA are injected as aqueous solutions in the microfluidic channels and the detection chamber respectively. After the injection, the solutions are dried at 85 °C for one hour in the oven. It should be noted that each chip is used just once, therefore it is disposable and there is no need for the S.A. and the NEDA replenishment. The ingredients are immobilized in their locations inside the channel before sealing of the chip with the upper PMMA layer.

### 2.3. Detection Setup

**Figure 3:**
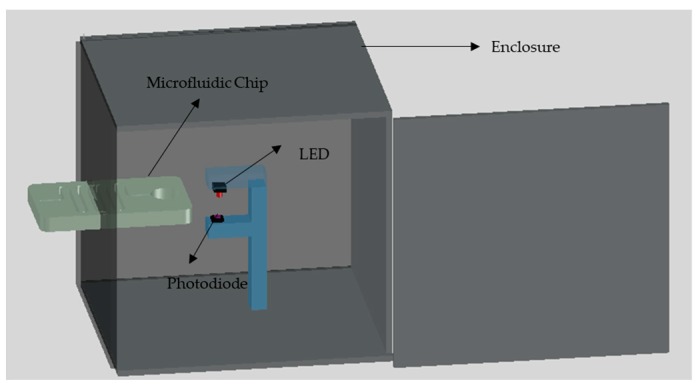
The developed setup of the nitrite detection system.

The main goal of this work is to develop a low-cost, simple and portable setup for the detection of chemical compounds such as nitrite. Therefore, a low-cost LOC setup is designed and developed in a simple laboratory environment where the developed colorimetric detection setup has the ability to provide the results of the test directly without the need for further procedures. The primary detection system is placed inside an 80 mm × 84 mm × 90 mm black PMMA box with 3 mm wall thickness where the front face of the box has a slot for the placement of the microfluidic chip, as shown in Figure 3. The primary setup consists of three components namely; green LED, photodiode and a microcontroller. The green LED which is obtained from Farnell, Aschheim, Germany can emit light with a wavelength in the range of 520 nm to 530 nm through the detection chamber. The photodiode which is purchased from Farnell, Aschheim, Germany is placed directly to face the LED where the detection chamber is placed in between. The color of the solution in the detection chamber affects the intensity of the light received by the photodiode, which affects the voltage signal output from the photodiode.

The output voltage signal from the photodiode is transferred to the microcontroller for processing. Arduino Mega is used in this work as a microcontroller where it is attached with liquid crystal display (LCD) shield to preview the detection results directly. It should be noted that the LED is powered from the Arduino without the need for additional power source. The voltage signal output from the photodiode is displayed on the LCD where the Arduino is programmed to display on average 20 readings each second where each test trial takes about 5 seconds which is the average of 100 readings. The intensity of the light transmitted to the photodiode through the detection chamber decreases as the concentration of nitrite increases, which in return decreases the corresponding voltage signal form the photodiode.

### 2.4. Nitrite Extraction from Processed Meat Samples

Five grams of processed meat are blended using a Moulinex blender for 30 s, then added to 50 mL of 1:1 water –phosphate buffer. The mixture is then ultrasonicated for 15 min and left to settle down for 10 min. The mixture is then filled in 15 mL corning centrifuge tubes and centrifuged at 3600 rpm for 10 min. Prior to filtration with C_18_ filter cartridges (Machery-Nagel, Düren, Germany) the filter cartridges are conditioned using 5 mL of methanol followed directly with 5 mL of distilled water. After the cartridges have been conditioned, the meat sample is filtered using the Machery Nagel C_18_ cartridges and vacuum manifold (JP Selecta, Barcelona, Spain). The overall sample preparation process takes about 30 min. Afterwards, the filtrated sample becomes ready for the detection process, which is explained in the next section.

### 2.5. Operational Concept

**Figure 4:**
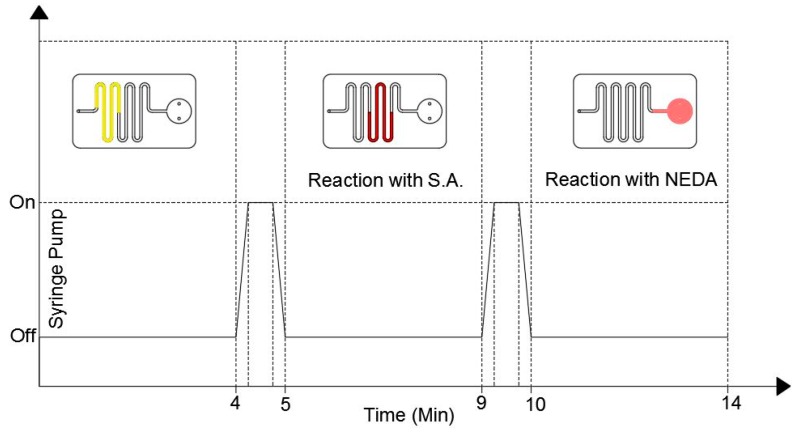
The duty cycle of the syringe pump (bottom part) and the fluid position inside the microfluidic chip during the detection process.

The proposed microfluidic chip design only requires pumping sources without the need for a valving system to start and control the chemical reaction in the coated channel and detection chamber. In this study a syringe pump is used to push the fluid to the detection chamber through the microfluidic channel with accurate flow rates. Figure 4 shows the duty cycle of the syringe pump as well as the location of the fluid during the pump cycle.

During the first 4 min the syringe pump is turned off where the fluid is located in the uncoated channels. After the first 4 min, the pump is turned on for one minute with a low accuracy flow rate to push the fluid to the S.A coated channel only. The pump is then turned off for 4 min to allow the fluid to react with the S.A. coating. After that, the pump is turned on for 1 min to push the fluid to the detection chamber where it is left there for 4 min to allow the reaction between the fluid and the NEDA coating. The color of the fluid turns to pink, which indicates that the reaction is over and the results can be taken where the intensity of the pink color is proportional to the nitrite concentration in the sample. The detection results can be then obtained by inserting the microfluidic chip into the detection setup.

## 3. Results and Discussion

### 3.1. Calibration Curve and the Statistical Parameters

Nitrite standard solutions with different concentrations are used in order to establish the calibration curve where the solutions are prepared by serial dilution from a stock with an initial concentration of 5 × 10^−4^ M. Table 3 shows the prepared solution and the corresponding absorbance values, which have been taken in sets of four trials.

The absorbance of the light is related to the analyte concentration via Beer-Lambert law which defines the relationship between the analyte absorbance and its concentration as;

(1) A = ε ×  b × c

where *A* is the absorbance, *b* is the path length in cm and *c* is the concentration in M. *ε* is the molar absorptivity and has the units M^−1^∙cm^−1^.

**Figure 5:**
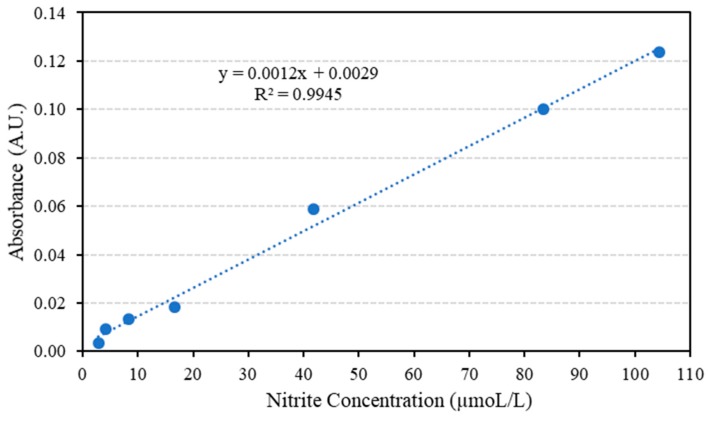
The average absorbance for nitrite solutions of various concentrations.

As shown in Table 3, the employed concentration range resides between the limits where there is linear adherence between concentration and absorbance. The limits are 1.00 µM and 0.0100 M, deviation from linearity is usually observed below 1.00 × 10^−6^ M due to scarcity of the absorbing species, as a consequence, negative deviation is observed. Above 1.00 × 10^−2^ M, the solution becomes opaque, therefore a significant portion of the incident light is reflected rather than being absorbed and negative deviation from linearity is reported [35]. Th e average absorbance and nitrite solution concentration (in ppm) are plotted to form the calibration curve as in Figure 5.

The concentration of nitrite in the minced meat samples is first found from the obtained correlation in Figure 5, then the concentration in mol/L is converted to ppm; a sample calculation is presented below to clarify the steps followed.

For Sharawi Extra Corned Beef, the reported absorbance for the very first trial is 0.02019 (Table A1). The corresponding concentration in molarity could be calculated from the linear equation that correlates absorbance to concentration in molarity as:

Concentration = (*A* − 0.0029)/1172.9

= (0.02019 − 0.0029)/1172.9 = 1.474 × 10^−5^ M

= 1.474 × 10^−5^ mol nitrite/L solution

The next step is the conversion of the calculated nitrite in molarity to the nitrite content in the meat sample in ppm which can be done as follow;

(1.474 × 10^−5^ moL nitrite/L solution) × (46.01g nitrite/1 moL nitrite) × (0.10 L solution/5.00 g meat) × (10^6^ µg nitrite/1 g meat) = 13.56 µg nitrite/1g meat = 13.56 ppm.

The correlation coefficient (R^2^), the limit of detection (LOD) and the limit of quantitation (LOQ) which are considered as the three key statistical parameters, are calculated in terms of absorbance and concentration using Beer’s Law. The LOD and LOQ can be calculated as:

(2) LOD = 3σ/m

(3) LOQ = 10σ/m

where *σ* is the standard deviation and *m* is the slope of the line that correlates the absorbance values to their corresponding concentrations. Table 4 shows the statistical parameters obtained.

The key improvement in this work when compared to the work published in the literature could be attributed to a combination of two factors, the absence of cadmium powder which is used for nitrate to nitrite reduction. That powder leaches through the channel and reduces the obtained sensitivity. The other factor is the solid phase extraction technique used in this study, which is responsible for the removal of most of the contaminants and interfering agents that may reduce the system ability to detect the nitrite. As a consequence, 6.3 times enhancement in the limit of detection is achieved with a LOD of 1.24 × 10^−2^ ppm [17].

The reported percentage standard deviation (5.698%) could be attributed to the utilization of a single beam setup. Usually absorbance measurements are performed using either single beam or double beam configuration, with preference for the double beam design since it is based on the continuous comparison of the signal obtained from the absorbing analyte and its blank or;

(4) $$A\;=\log\left(P_{o}/P\right)\;=\log\left(V_{o}/V\right)$$

where *P_o_* and *P* are power intensities of the signals transmitted from the blank (analyte free solution) and the analyte solution, respectively. As a consequence, any source fluctuations (from the LED in this study) will affect both signals to the same extent. In single beam devices, the analyte signal is compared with that taken at beginning of the spectrometric measurements, therefore, any disturbance in the radiant intensity could not be accounted for, during course of the measurements.

### 3.2. Nitrite content detected in meat sample using Lab on Chip (LOC)

The nitrite content of seven different processed meat samples purchased from the local market is evaluated based on the obtained calibration curve where each sample is tested 10 times as shown in Appendix A. Table 5 shows the average nitrite concentration in the tested samples. It can be concluded from Table 5 that the nitrite levels in all of the tested samples are within the acceptable range (below 150 ppm) and so the consumption of the analyzed processed meat samples is safe and has no negative impact on human health.

As shown in Table 5, the detected nitrite content varies from 5.89 ppm to 19.04 ppm. Variation in the obtained numerical values not only depends on amount of the nitrite added during course of the curing, but also on nature of the analyzed meat and the extraction approach. Practically, solid phase extraction of NO_2_^−^ from dehydrated samples such as salami was more convenient than that of the rest of the sample because of the low moist content, therefore, well chopped meat samples were obtained from mincing of the salami sample, that is then sonicated and treated according to the procedure mentioned in the experimental section. As a consequence, almost all of the nitrite content could be extracted from the salami sample. In this context, it is worth it to mention that salami is usually cured with a relatively high amount of sodium nitrite to elongate its shelf life since it is usually kept at ambient conditions.

## 4. Conclusions and Future Work

Nitrite has been used as an additive to plenty of products in the last few centuries as a preservative and as flavor enhancer, however the consumption of excess amount of nitrite causes severe health issues for humans. Therefore, the measurement of nitrite concentration in human goods using a low-cost setup is vital. In this work, a homemade lab on a chip setup is designed and fabricated to measure the nitrite concentration in various meat samples. The developed detection device is characterized by its low-cost, effectiveness, small size and portability, which makes it suitable for measurements conducted in remote research areas and refugee camps. Moreover, a major improvement over the previous studies is presented in this work where the nitrite detection is carried out without the use of cadmium powder in addition to the solid phase extraction technique for the meat samples, which enhanced the limit of detection achieved by more than 6 times. The developed setup is used to test the nitrite concentration in the processed meat available in the market, where the results show that all the tested subjects had nitrite concentrations in the safe range and are suitable for human consumption.

For future work, the device can be further developed into a two-beam measuring device so that the blank and sample absorbance are measured simultaneously instead of first measuring the blank absorbance followed by the sample absorbance.

## Figures and Tables

**Table 1:** Detection of nitrite in food and biological samples using different systems.

System	LOD in ppm	Samples	Ref.
PMMA multichannel microfluidic disc	0.00920 × 10^−2^	Natural water samples taken from Southampton waters	[14]
Acrylic dipstick	N.A.	Urine samples	[25]
µPaper Analytical Device	4.60 × 10^−2^	Wide range of biological and food samples; meat, saliva, ham, water, etc.	[26]
PMMA single channel chip	7.82 × 10^−2^	Mineral and tap waters	[17]
Present	1.24 × 10^−2^	meat products	

**Table 2:** The chemicals used in the nitrite detection in this study.

Chemical Name	Source	Country
Potassium nitrite	Sigma Aldrich	St. Louis, MO, USA
Dihydrogen sodium phosphate	Sigma Aldrich	St. Louis, MO, USA
Phosphoric acid	Honeywell-Riedel de Haen	Morristown, NJ, USA
Benzensulfanylamide (S.A.)	Applichem GmbH	Darmstadt, Germany
N-1-naphthylethylenediamin dihydrochloride (NEDA)	Carlo Erba reagents	Peypin, France
Potassium hydroxide	S.D. Fine Chem Limited	Mumbai, India
Nafion	Sigma Aldrich	St. Louis, MO, USA
Ethanol	Tedia	Fairfeild, OH, USA
Methanol	Tedia	Fairfeild, OH, USA

**Table 3:** The absorbance and average absorbance for nitrite solutions of different concentrations which were used to form the calibration curve.

Concentration (moL/L)	Average Absorbance (*N* = 4)	Standard Deviation of the Absorbance	STDEV/Average Abs. (%)
2.86 × 10^−6^	3.645 × 10^−3^	1.063 × 10^−4^	2.916
4.17 × 10^−6^	9.083 × 10^−3^	4.156 × 10^−4^	4.575
8.34 × 10^−6^	0.0134	4.349 × 10^−4^	3.241
1.67 × 10^−5^	0.0184	8.762 × 10^−4^	4.750
4.17 × 10^−5^	0.0588	8.974 × 10^−4^	1.525
8.34 × 10^−5^	0.1002	10.008 × 10^−4^	0.998
1.04 × 10^−4^	0.1234	21.668 × 10^−4^	1.755

**Table 4:** The reported statistical parameters.

Absorbance for Blank	Average Absorbance of the Blank (*N* = 4)	Standard Deviation of the Blank Absorbance	STDEV/Average Abs. (%)	LOD (ppm)	LOQ (ppm)
9.00 × 10^−5^	9.22 × 10^−5^	5.252 × 10^−6^	5.698	0.0124	0.0412
1.00 × 10^−4^					
9.00 × 10^−5^					
8.87 × 10^−5^					

**Table 5:** The nitrite detected in various meat samples using the Lab on Chip (LOC).

Brand	Average [NO_2_^−^] (ppm)	STDEV [NO_2_^−^] (ppm)
Toulkarem Roast Chicken Breast	8.82	0.474
Siniora Roast Beef Shoulder	10.79	0.168
Siniora Pastrami	13.59	0.281
Siniora Salami	19.04	0.200
Siniora Italian Roast Beef	5.89	0.218
Zwan Chicken Luncheon Meat	6.83	0.296
Sharawi Extra Corned Beef	14.77	0.774

## Appendix A

**Table A1:** The absorbance and the nitirite concentration in the tested meat samples.

Brand	Trial	Absorbance	[NO_2_^−^] (M)	[NO_2_^−^] (ppm)
Toulkarem Roast Chicken Breast	1	0.01346	9.003 × 10^−6^	8.28
	2	0.01452	9.907 × 10^−6^	9.12
	3	0.01383	9.319 × 10^−6^	8.58
	4	0.01392	9.396 × 10^−6^	8.65
	5	0.01376	9.259 × 10^−6^	8.52
	6	0.01373	9.234 × 10^−6^	8.50
	7	0.01379	9.285 × 10^−6^	8.54
	8	0.01407	9.523 × 10^−6^	8.76
	9	0.01514	1.044 × 10^−5^	9.60
	10	0.01519	1.048 × 10^−5^	9.64
Siniora Roast Beef Shoulder	1	0.01694	1.197 × 10^−5^	11.02
	2	0.01701	1.203 × 10^−5^	11.07
	3	0.01687	1.191 × 10^−5^	10.96
	4	0.01665	1.172 × 10^−5^	10.79
	5	0.01658	1.166 × 10^−5^	10.73
	6	0.01659	1.167 × 10^−5^	10.74
	7	0.01652	1.161 × 10^−5^	10.69
	8	0.01641	1.152 × 10^−5^	10.60
	9	0.01647	1.157 × 10^−5^	10.65
	10	0.01645	1.155 × 10^−5^	10.63
Siniora Pastrami	1	0.02015	1.471 × 10^−5^	13.53
	2	0.02023	1.478 × 10^−5^	13.60
	3	0.02120	1.560 × 10^−5^	14.36
	4	0.02023	1.478 × 10^−5^	13.60
	5	0.02022	1.477 × 10^−5^	13.59
	6	0.02009	1.466 × 10^−5^	13.49
	7	0.01997	1.455 × 10^−5^	13.39
	8	0.02002	1.460 × 10^−5^	13.43
	9	0.02007	1.464 × 10^−5^	13.47
	10	0.01999	1.457 × 10^−5^	13.41
Siniora Salami	1	0.02745	2.093 × 10^−5^	19.26
	2	0.02747	2.095 × 10^−5^	19.28
	3	0.02745	2.093 × 10^−5^	19.26
	4	0.02735	2.085 × 10^−5^	19.18
	5	0.02719	2.071 × 10^−5^	19.06
	6	0.02707	2.061 × 10^−5^	18.96
	7	0.02696	2.051 × 10^−5^	18.88
	8	0.02683	2.040 × 10^−5^	18.77
	9	0.02705	2.059 × 10^−5^	18.95
	10	0.02682	2.039 × 10^−5^	18.77
Siniora Itialian Roast Beef	1	0.00744	6.343 × 10^−6^	5.84
	2	0.00714	6.087 × 10^−6^	5.60
	3	0.00769	6.556 × 10^−6^	6.03
	4	0.00763	6.505 × 10^−6^	5.99
	5	0.00695	5.925 ×10^−6^	5.45
	6	0.00767	6.539 × 10^−6^	6.02
	7	0.00747	6.369 × 10^−6^	5.86
	8	0.00752	6.411 × 10^−6^	5.90
	9	0.00765	6.522 × 10^−6^	6.00
	10	0.00790	6.735 × 10^−6^	6.20
Zwan Chicken Luncheon Meat	1	0.01114	7.025 × 10^−6^	6.46
	2	0.01142	7.264 × 10^−6^	6.68
	3	0.01190	7.673 × 10^−6^	7.06
	4	0.01117	7.051 × 10^−6^	6.49
	5	0.01157	7.392 × 10^−6^	6.80
	6	0.01227	7.989 × 10^−6^	7.35
	7	0.01152	7.349 × 10^−6^	6.76
	8	0.01170	7.503 × 10^−6^	6.90
	9	0.01207	7.818 × 10^−6^	7.19
	10	0.01131	7.170 × 10^−6^	6.60
Sharawi Extra Corned Beef	1	0.02019	1.474 × 10^−5^	13.56
	2	0.02068	1.516 × 10^−5^	13.95
	3	0.02131	1.570 × 10^−5^	14.44
	4	0.02161	1.595 × 10^−5^	14.68
	5	0.02294	1.709 × 10^−5^	15.72
	6	0.02202	1.630 × 10^−5^	15.00
	7	0.02077	1.524 × 10^−5^	14.02
	8	0.02286	1.702 × 10^−5^	15.66
	9	0.02288	1.703 × 10^−5^	15.68
	10	0.02194	1.623 × 10^−5^	14.94
